# Anterior surgical treatment for cervical degenerative radiculopathy: a prediction model for non-success

**DOI:** 10.1007/s00701-022-05440-2

**Published:** 2022-12-08

**Authors:** Christer Mjåset, Tore K. Solberg, John-Anker Zwart, Milada C. Småstuen, Frode Kolstad, Margreth Grotle

**Affiliations:** 1 Faculty of Medicine, University of Oslo, Oslo, Norway; 2 Department of Neurosurgery, Oslo University Hospital, Oslo, Norway; 3 Division of Clinical Neuroscience, Department of Research and Innovation, Oslo University Hospital, P.O. Box 4956, 0424 Oslo, Nydalen Norway; 4 Institute of Clinical Medicine, The Arctic University of Norway, Tromsø, Norway; 5 Department of Neurosurgery and The Norwegian Registry for Spine Surgery (NORspine), The University Hospital of North Norway, Tromsø, Norway; 6 Department of Rehabilitation and Technology, Faculty of Health Science, Oslo Metropolitan University, St. Olavs Plass, P.O. Box 4, 0130 Oslo, Norway

**Keywords:** Degenerative neck surgery, Predictors, Prognostic model, Outcome, Neck disability, Arm pain

## Abstract

**Purpose:**

By using data from the Norwegian Registry for Spine Surgery, we wanted to develop and validate prediction models for non-success in patients operated with anterior surgical techniques for cervical degenerative radiculopathy (CDR).

**Methods:**

This is a multicentre longitudinal study of 2022 patients undergoing CDR surgery and followed for 12 months to find prognostic models for non-success in neck disability and arm pain using multivariable logistic regression analysis. Model performance was evaluated by area under the receiver operating characteristic curve (AUC) and a calibration test. Internal validation by bootstrapping re-sampling with 1000 repetitions was applied to correct for over-optimism. The clinical usefulness of the neck disability model was explored by developing a risk matrix for individual case examples.

**Results:**

Thirty-eight percent of patients experienced non-success in neck disability and 35% in arm pain. Loss to follow-up was 35% for both groups. Predictors for non-success in neck disability were high physical demands in work, low level of education, pending litigation, previous neck surgery, long duration of arm pain, medium-to-high baseline disability score and presence of anxiety/depression. AUC was 0.78 (95% CI, 0.75, 0.82). For the arm pain model, all predictors for non-success in neck disability, except for anxiety/depression, were found to be significant in addition to foreign mother tongue, smoking and medium-to-high baseline arm pain. AUC was 0.68 (95% CI, 0.64, 0.72).

**Conclusion:**

The neck disability model showed high discriminative performance, whereas the arm pain model was shown to be acceptable. Based upon the models, individualized risk estimates can be made and applied in shared decision-making with patients referred for surgical assessment.

## Introduction

Cervical degenerative radiculopathy (CDR) is caused by nerve root compression by a herniated or bulging disc and/or ligament hypertrophy and bony spurs. The incidence rate is reported to be approximately 80 per 100,000 people [32], and surgical treatment is usually offered to patients with persistent arm pain and/or paresis [10]. With the introduction of modern operative techniques like anterior cervical discectomy and fusion or disc arthroplasty, treatment safety and effectiveness have increased dramatically [10]. Currently, day surgery is practiced in many clinics worldwide [14, 20]. Still, far from all patients improve after surgery [5–7, 12]. Many studies have investigated what predicts a beneficial outcome [21, 31], but there is current lack of evidence concerning factors associated with unfavourable or non-successful outcomes. A high body mass index [47], mental health problems [1, 19] and lower social class [15] are individual patient characteristics that have been linked to poor treatment outcomes after cervical degenerative surgery. Predictive models can aid in calibrating surgeons’ and patients’ expectations prior to intervention, thus enhancing clinical decision-making and patient selection for surgical intervention.

The primary objective of this study was to develop and validate a prediction model for non-success in neck disability 12 months after surgery for CDR. Secondary objectives were to provide the same analysis for arm pain and to develop a risk matrix for the primary outcome to exemplify the use of the model in a clinical setting.

## Methods

### Design and ethics

This is a multicentre longitudinal study following the recommendations for reporting in observational studies, STROBE criteria [44] and the methodological framework proposed by the PROGRESS framework [33, 40]. The manuscript is reported according to the Transparent Reporting of a multivariable prediction model for Individual Prognosis Or Diagnosis (TRIPOD) guidelines [9]. We used the Prediction model Risk Of Bias Assessment Tool (PROBAST) to minimize the risk of bias [27, 45]

Our research protocol was approved by the Norwegian Committee for Medical and Health Research Ethics Midt (2014/344). Written informed consent was obtained from all patients.

### Patients and surgical treatment

Data from the Norwegian Registry for Spine Surgery (NORspine) from 2011 to 2016 was used. NORspine is a government-funded comprehensive clinical registry receiving no industry funding and used for quality assessment and research. Informed consent is obtained from all patients before they enter the registry. Currently, all centres performing cervical spine surgery in Norway report data to NORspine (coverage = 100%), and the operation recording rate is 78% (completeness) [3]. Patients who had undergone anterior cervical discectomy and fusion or arthroplasty surgery due to cervical degenerative radiculopathy in the period were included. For both groups, baseline characteristics and 12-month outcome data were similar, except from baseline Neck Disability Index and neck pain scores, which were slightly higher in the arthroplasty group (*p* = 0.02 and *p* = 0.002, respectively). Also, arthroplasty patients were operated in significantly lower number of levels (*p* < 0.001). Patients undergoing posterior cervical procedures due to CDR, as well as all patients operated for myelopathy symptoms, were excluded. Patients operated for tumours, fractures and primary infections are not included in NORspine.

Patients completed data at admission for surgery (baseline), after 3 and 12 months. Surgeon’s forms containing information about diagnosis, treatment and comorbidity were completed during the hospital stay. Only cohort participants responding to the 12-month questionnaire were included in the present study. Follow-up was conducted by the central registry unit without involvement of the treating hospital. The patients responded by questionnaires sent and returned by mail. One reminder with a new form was sent to non-respondents within 2 weeks.

### Outcome definitions

For the primary outcome, the Neck Disability Index (NDI) (0–100), non-success was defined as an absolute score of > 26 at 12-month follow-up. For the secondary outcome, arm pain intensity assessed by numerical rating scale (NRS-AP) (0–10), non-success was defined as a score ≥ 3 at 12-month follow-up. These estimates are based on a previous study for patients undergoing CDR surgery in Norway [25].

### Candidate predictors

Candidate predictors for non-success were selected from the comprehensive NORspine questionnaire administered before surgery, which consists of information about sociodemographic factors, lifestyle, work and clinical variables in addition to patient-reported outcome measures (PROMs). Data from the surgeons’ forms were used for information about diagnosis, treatment, comorbidity, the American Society of Anaesthesiologists physical status (ASA), surgical indication and type of operation. The selection of the final set of predictors was made after a thorough literature review where we identified the factors that have been found to be significantly and consistently associated with outcomes after CDR surgery.

The following predictors were selected for the model: gender, age groups (below 40 years, between 40 and 60 years or above 60 years), work status prior to operation (on sick leave, retired or disabled; on rehabilitation, out of work or on work return training; student, fully working or housewife/househusband), physical demands in work (working with computers, sitting, light physical work or hard physical work), educational level (high school or less, less than 4 years university or 4 or more years of university), mother tongue (Norwegian or non-native speaker), pending litigation (yes/no) (pending litigation defined as unresolved claims or litigation issues against the Norwegian Public Welfare Agency Fund concerning permanent disability pension or compensation claims against private insurance companies or the public Norwegian System of Compensation to Patients), duration of arm pain (less than 3 months, 3 to 12 months or more than 12 months), duration of pre-operative paresis (no paresis, less than 3 months or more than 3 months), body mass index (BMI) (equal to or below 30 or above 30), smoking (yes/no), comorbidities (yes/no), previous neck surgery (yes/no), number of surgical levels (one or more than one), daily use of analgetic drugs (yes/no), ASA level (level 1–2 or level 3–4), arm pain neck pain ratio (above 1 or below or equal to 1) [30] and anxiety/depression by the item on the EuroQol-5D-3L (EQ-5D) questionnaire (“moderate” to “extremely” anxious or depressed or “not anxious or depressed”). In addition, baseline outcome scores were included as potential predictors; the baseline scores were categorized into low, medium and high by percentile distribution.

### Sample size considerations

Since no consensus on sample size in prognostic modelling exists, we chose to follow the recommendations by Steyerberg [38]: (a) aiming for at least 100 events as a minimum for reliable estimation of the average risk and (b) aiming for at least 10 events per variable (EPV) and preferably 20, for reliable prediction modelling if the event rate is < 20% and higher EPV values if the event rate is between 20 and 80%. In the present material, approximately 700 cases had non-success at 12-month follow-up, and with this large number of EPV, we had nearly 40 cases per event and good statistical power for the prediction model analyses. The large EPV will reduce the potential for overfitting and optimism of the final models. Overfitting is defined as fitting a statistical model with too many effective degrees of freedom in the modelling process. Estimation bias is defined as the overestimation of effects of predictors because selection of the effects withstood a statistical test, whereas optimism is defined as the difference between true performance (performance in the underlying population, e.g. external validation sample) and apparent performance (development sample) [38].

### Statistical analysis

Statistical analyses were performed using IBM SPSS Statistics version 26 for Windows and the STATA version 16 for Windows. Missing data was checked for all variables and are reported together with descriptive data. Frequencies were used for categorical data and mean and standard deviation (SD) for continuous data. Continuous variables, such as baseline disability, were categorized to be adapted into a risk matrix. The distribution of baseline and 12-month scores of the two outcome measures are presented by mean scores and SD.

First, a univariable analysis of the candidate predictors was performed to assess the crude association between each candidate predictors and the two outcomes. Associations between outcomes and predictors are expressed as odd ratios (OR) with a 95% confidence interval (CI). Predictors reaching *p* < 0.1 in these analyses were entered into two multivariable logistic regression models (for primary and secondary outcome), where a stepwise backward elimination method was used. Variables that were not statistically significant (*p* > 0.05) in the multivariable models were removed from the model. The performance of the two final models was evaluated with (1) the explained variance by Nagelkerke’s *R*^2^, (2) the Hosmer–Lemeshow test *p* > 0.05) and (3) the discriminative ability of the model (the likelihood that the model allocates higher predicted risks to patients who achieve non-substantial improvement and lower predicted risks to those who do not) assessed by calculating the area under the receiver operating curves (AUC), also often referred to as the c-index [41]. The larger the AUC, the greater is the discriminative ability of the model. The discriminative performance of the models was considered acceptable if the AUC was ≥ 0.7 and good if the AUC was ≥ 0.8 (the c-criterion).

Internal validation was conducted by a bootstrap procedure (1000 samples) to estimate the amount of optimism in the two final models [26, 39]. A slope value was calculated (the closer to 1.0, the less over-optimism) and used to correct and shrink the regression coefficients, the *R*^2^ and the c-index.

### Clinical usefulness (risk matrix)

We assessed the potential clinical utility of the final prediction model for non-success in neck disability by developing a risk matrix for two hypothetical patient case profiles with few and many predictors present. Regression coefficients from the final disability model were converted into probabilities, and a risk score for each of the two individual case profiles was calculated by the sum of the products of individual values of each predictor variable and its regression coefficient. Depending on the presence or absence of the risk factors, the matrix was then calculated as probability for a non-substantial improvement after 12 months for each of the patients.

## Results

There were 3142 patients who had undergone either anterior discectomy and fusion (3109) or arthroplasty (33) due to CDR during the study period. Out of these, 2022 (64.4%) completed 12-month follow-up and were included in the analyses (2020 for the NDI analysis and 1980 for the NRS-AP analysis). Compared to responders at 12-month follow-up, non-responders were less likely to be female, significantly younger, had higher neck disability, more neck pain, lower quality of life, were less educated and more likely to be smoking.

Demographic and clinical characteristics at baseline for the included participants are summarized in Table 1, including the missing values for each variable. Gender distribution was equal (50%), and the average age at baseline was 51 years. Most patients were on sick leave before surgery. Approximately 40% of the patients reported that their job involved hard physical work, and only 17% had high level of education. Nearly half of the patients had experienced neck pain for more than 1 year. Only 5% of the patients had an ASA level of 3 or more prior to surgery. There were few missing values for the candidate predictor variables, except for physical demands in work, pending litigation, duration of pre-operative paresis, previous neck surgery and arm/neck pain ratio (Table 1).

**Table 1:** Characteristics of participants at baseline (*n*=3142) and 12-months follow-up (2022), including number of missing values in each of the variables

Characteristics and domain	Baseline (*n*=3142)	Complete sample (*n*=2022)
Sociodemographic		
Female gender, *n* (%)	1502 (47.8)	1005 (49.7)
Age, mean years (SD)	49.5 (9.3)	51.0 (9.2)
Age <40	404 (12.9)	189 (9.4)
Age 40-60	2355 (75.0)	1534 (75.9)
Age >60	380 (12.1)	298 (14.7)
Missing	3 (0)	1 (0)
Work status prior to operation, *n* (%)		
Student, in work or at home	974 (31.4)	187 (9.3)
Retired or disability pension	460 (14.8)	334 (16.6)
Rehabilitation pension	278 (9.0)	157 (7.8)
Sick leave	1390 (44.8)	1333 (66.3)
Missing	40 (1.3)	11 (0.5)
Physical demands in work, *n* (%)		
Working in front of a computer/sitting still	1039 (38.8)	712 (41.6)
Light physical work	486 (18.1)	319 (18.6)
Hard physical work	1156 (43.1)	681 (39.8)
Missing	461 (14.7)	310 (15.5)
Educational level, *n* (%)		
High school or less	1963 (64.1)	1231 (62.3)
Less than 4 years of university	626 (20.5)	405 (20.5)
4 or more years of university	472 (15.4)	339 (17.2)
Missing	81 (2.5)	47 (2.3)
Non-native speaker, *n* (%)	229 (7.3)	134 (6.6)
Missing	2 (0)	2 (0)
Pending litigation^1^, *n* (%)		
No	2764 (89.0)	1680 (83.1)
Yes	342 (11.0)	342 (16.9)
Missing	36 (1.1)	0 (0)
Physical/somatic		
Obesity (Body Mass Index ≥ 30), *n* (%)	657 (21.4)	419 (21.2)
Missing	78 (2.5)	48 (2.4)
Smoking	1043 (33.9)	582 (29.4)
Missing	63 (2.0)	40 (2.0)
Comorbidity	1272 (40.5)	842 (41.6)
Missing	0 (0)	0 (0)
Previous neck surgery (same level)	265 (8.6)	160 (8.1)
Missing	52 (1.7)	40 (2.0)
Number of surgical levels		
One level	2347 (75.3)	1488 (74.4)
Two or more levels	768 (24.7)	512 (25.6)
Missing	27 (0.9)	22 (1.0)
Type of surgery		
Anterior discectomy and fusion	3109 (98.9)	2003 (99.0)
Anterior discectomy and arthroplasty	33 (1.1)	19 (1.0)
Missing	0 (0)	0 (0)
Clinical self-report		
Duration of arm pain		
< 3 months	440 (14.4)	284 (14.4)
3-12 months	1120 (36.7)	714 (36.2)
> 1 year	1494 (48.9)	975 (49.4)
Missing	88 (2.8)	49 (2.4)
Duration of pre-operative paresis		
No paresis	641 (22.6)	425 (22.4)
< 3 months	450 (15.8)	357 (18.8)
3 months or more	1750 (61.6)	1116 (58.8)
Missing	301 (9.6)	124 (6.1)
Daily use of analgetic drugs (vs < daily use)	1634 (52.8)	1042 (52.4)
Missing	48 (1.5)	25 (1.2)
ASA level of 3 or more^2^	151 (5.0)	101 (5.2)
Missing	99 (3.2)	76 (3.8)
Arm pain worse than neck pain	945 (32.0)	591 (31.2)
Missing	187 (6.0)	125 (6.1)
Baseline neck pain (NRS^3^), mean (SD)	6.1 (2.5)	6.1 (2.5)
Baseline arm pain (NRS^3^), mean (SD)	6.4 (2.4)	6.4 (2.4)
Baseline disability (NDI^4^), mean (SD)	41.6 (15.1)	41.0 (15.2)
Psychological		
Anxiety or depression^5^	1355 (44.0)	832 (41.8)
Missing	62 (2.0)	31 (1.0)

^1^Pending medical claim/litigation against the Norwegian public welfare agency fund concerning disability pension or pending medical compensation claim/litigation against private insurance companies or the public Norwegian System of Compensation to Patients. ^2^American Society of Anesthesiologists grade. ^3^Numeric rating scale (0-10). ^4^Neck Disability Index, 0-100 (no-maximal disability). ^5^Based on scoring “moderate” or “extremely” anxious or depressed in the item in EQ-5D-3L questionnaire

The mean scores of the NDI and NRS arm pain at 12-month follow-up was 23.4 (SD 18.8) and 2.9 (SD 2.8), respectively. A total of 38.0% had non-successful outcomes in neck disability and 35.3% in arm pain.

Table 2 presents the univariable analysis of all candidate predictors. Most candidate predictors showed a statistical univariate relationship to the two outcomes: female gender, being retired or receiving disability or rehabilitation pension, high physical demands in work, low education level, being a non-native speaker, having a pending litigation, smoking, presence of comorbidity, having undergone previous neck surgery, having long duration of arm pain or long duration of paresis prior to surgery, high ASA level, daily use of analgetic drugs, arm pain worse than neck pain, presence of anxiety/depression or high baseline scores of NDI or arm pain. Age, obesity and number of surgical levels were not significantly associated to any of the two outcomes.

**Table 2:** Univariate associations at 12-month follow-up between candidate predictors and the two outcomes; non-substantial improvement in disability and arm pain. Regression coefficient and odds ratio (95% confidence intervals) (*n*=2022)

	Total number of cases	Non-success in neck disability (12-mo NDI ≥26) *	Odds Ratio (95% CI)	Total number of cases	Non-success in arm pain (12-mo arm pain ≥3) **	Odds Ratio (95% CI)
	*n*=2020	*n*=768 (38%)		*n*=1980	*n*=698 (35.3%)	
Socio-demographic						
Female gender	2020	408	1.25 (1.04, 1.49)	1980	366	1.18 (0.98, 1.42)
Age, years	2019			1979		
Age <40		69	Ref		59	Ref
Age 40-60		593	1.09 (0.79, 1.49)		549	1.23 (0.89, 1.70)
Age >60		105	0.94 (0.65, 1.38)		89	0.99 (0.67, 1.49)
Work status	2009			1969		
Student, in work or stay-at-home		61	Ref		62	Ref
Retired or disability pension		173	2.25 (1.55, 3.27)		134	1.41 (0.96, 2.05)
Rehabilitation pension		113	5.31 (3.31, 8.43)		95	3.20 (2.04, 5.00)
Sick leave		415	0.93 (0.67,1.30)		403	0.86 (0.62, 1.20)
Physical demands in work	1712			1683		
Computers/ sitting		187	Ref		174	Ref
Light physical work		103	1.34 (1.00, 1.79)		99	1.41 (1.05, 1.89)
Hard physical work		296	2.16 (1.72, 2.70)		286	2.28 (1.81, 2.87)
Educational level	1974			1935		
High school or less		528	Ref		486	Ref
Less than 4 years of university		143	0.73 (0.58, 0.92)		129	0.7 (0.55, 0.89)
4 or more years of university		78	0.40 (0.30, 0.52)		61	0.33 (0.25, 0.45)
Non-native speaker	2018	70	1.08 (1.03, 1.13)	1978	72	1.11 (1.06, 1.16)
Pending litigation^1^	1996			1957		
None		565	Ref		537	Ref
Yes		119	3.06 (2.26, 4.15)		95	2.01 (1.49, 2.72)
Already approved		71	3.13 (2.12, 4.60)		59	2.27 (1.55, 3.33)
Physical/ somatic						
Obesity (Body Mass Index ≥ 30)	1972	169	1.14 (0.92, 1.42)	1932	141	0.98 (0.78, 1.24)
Smoking	1980	273	1.71 (1.40, 2.08)	1941	263	1.93 (1.58, 2.36)
Comorbidities	2020	378	1.66 (1.38, 1.99)	1980	323	1.55 (1.12, 1.63)
Previous neck surgery	1980	89	1.09 (1.05, 1.15)	1940	84	1.10 (1.06, 1.14)
Number of surgical levels	1998			1958		
One level		550	Ref		512	Ref
Two or more levels		209	1.18 (0.96, 1.45)		177	0.91 (0.82, 1.25)
Pain and symptoms						
Duration of arm pain	1971			1933		
< 3 months		65	Ref		57	Ref
3-12 months		237	1.66 (1.21, 2.29)		214	1.67 (1.20, 2.33)
> 1 year		444	2.81 (2.07, 3.81)		406	2.83 (2.06, 3.90)
Duration of pre-operative paresis	1828			1860		
No paresis		124	Ref		107	Ref
< 3 months		110	1.08 (0.80, 1.47)		96	1.08 (0.78, 1.49)
3 months or more		467	1.75 (1.38, 2.23)		441	1.91 (1.49, 2.46)
Daily use of Analgetic drugs	1985	268	0.45 (0.37, 0.54)	1946	269	0.58 (0.48, 0.71)
ASA level 3 or more^2^	1944	47	1.50 (1.00, 2.24)	44	44	1.57 (1.05, 2.37)
Neck pain worse than arm pain	1895	270	1.13 (1.08, 1.17)	1951	195	0.88 (0.72, 1.08)
Baseline NDI score (0-100)^3^	2013			1973		
Low (0-40)		173	Ref		225	Ref
Medium (41-60)		448	4.84 (3.92, 5.99)		358	2.29 (1.87, 2.28)
High (> 60)		144	10.74 (7.62, 15.14)		113	4.21 (3.06, 5.80)
Baseline NRS arm pain (0-10)^4^	1982			1944		
Low (0-5)		117	Ref		86	Ref
Medium (6-7)		300	1.45 (1.12, 1.87)		273	1.87 (1.42, 2.48)
High (> 8)		334	2.06 (1.59, 2.67)		330	3.01 (2.28, 3.40)
Psychological						
Anxiety or depression^5^	1989	422	2.62 (2.18, 3.16)	1950		1.75 (1.45, 2.11)

*38% did not achieve a substantial improvement in disability. **35.3% did not achieve a substantial improvement in arm pain.^1^Pending medical claim/litigation against the Norwegian public welfare agency fund concerning disability pension or pending medical compensation claim/litigation against private insurance companies or the public Norwegian System of Compensation to Patients.^2^American Society of Anesthesiologists grade. ^3^Neck Disability Index, 0-100 (no-maximal disability). ^4^Numeric Rating Scale (0-10). ^5^Based on scoring “moderate” to “extremely” anxious or depressed in the item in EQ-5D-3L questionnaire

**Fig. 1:**
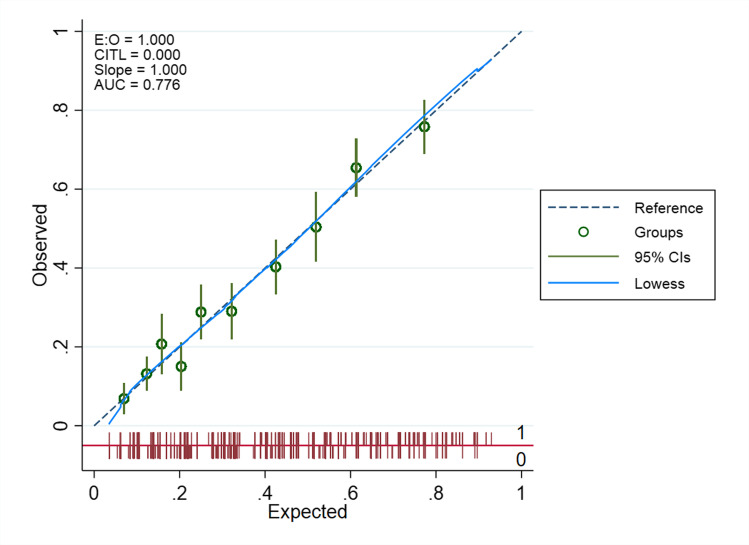
Calibration plot for the final model predicting no substantial improvement in neck disability at 12-month follow-up (E:O, expected/observed; CITL, calibration-in-the-large; slope, calibration slope; AUC, area under the curve; CIs, confidence intervals)

**Fig. 2:**
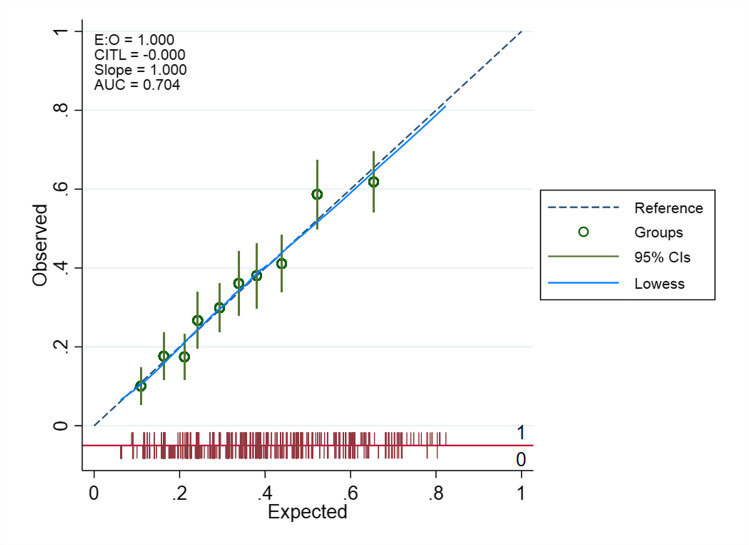
Calibration plot for the final model predicting no substantial improvement in arm pain at 12-month follow-up (E:O, expected/observed; CITL, calibration-in-the-large; slope, calibration slope; AUC, area under the curve; CIs, confidence intervals)

Table 3 shows the results from the multivariable analyses. Seven predictors (hard physical demands in work, low level of education, pending litigation, previous neck surgery, duration of arm pain > 3 months, medium or high levels of baseline disability and anxiety/depression) showed statistically significant association with non-success in neck disability. The model displayed good overall performance with Nagelkerke *R*^2^ of 28.3%, non-significant Hosmer–Lemeshow test and AUC 0.78 (95% CI 0.75, 0.82). The prediction model for non-success in arm pain included six of the same predictors (hard physical demands in work, low level of education, pending litigation, previous neck surgery, duration of arm pain > 3 months, medium or high levels of baseline disability) in addition to foreign mother tongue, smoking and medium or high levels of arm pain. This model showed acceptable performance with Nagelkerke *R*^2^ of 15.5% and AUC of 0.68 (95% CI 0.64, 0.72). The calibration plots for the two models are displayed in Figs. 1 and 2. Both models had high calibration slopes of 1.0, indicating no overfitting of the models.

**Table 3:** Predictors for non-success in neck disability or arm pain at 12-months after surgery. Results are presented by Odds Ratio (OR) and bootstrapped 95% Confidence Intervals (CI) for the significant variables

	Non-success in neck disability (12-mo NDI^1^≥26*) *n*=1593	Non-success in arm pain (12-mo NRS-AP^2^≥3**) *n*=1546
Hard physical demands in work (vs computers/sitting still or light physical work)	1.56 (1.22, 2.00)	1.46 (1.15, 1.85)
High educational level (4 or more years of university) (vs high school or less than 4 years of university)	0.57 (0.41, 0.78)	0.51 (0.36, 0.71)
Pending litigation^3^ (vs none)	2.38 (1.70, 3.34)	1.68 (1.21, 2.33)
Previous neck surgery (vs not)	2.52 (1.61, 3.96)	2.01 (1.33, 3.03)
Duration of arm pain		
< 3 months	Ref	Ref
3-12 months	1.81 (1.24, 2.65)	1.48 (1.01, 2.18)
> 1 year	2.51 (1.72, 3.66)	2.42 (1.66, 3.52)
Anxiety or depression	1.74 (1.38, 2.19)	-
Baseline NDI^1^ score		
Low (0-40)	Ref	Ref
Medium (41-60)	4.20 (3.22, 5.48)	1.79 (1.40, 2.29)
High (> 60)	7.79 (5.07, 11.98)	2.53 (1.67, 3.85)
Baseline Arm Pain score		
Low (0-4)	-	Ref
Medium (5-7)	-	1.66 (1.18, 2.32)
High (8-10)	-	2.04 (1.43, 2.90)
Foreign mother tongue (vs Norwegian)	-	1.71 (1.12, 2.61)
Smoking (vs no smoking)	-	1.44 (1.11, 1.85)
Nagelkerke R square	28.3%	17.3%
Discrimination, AUC^4^	0.78 (0.75, 0.82)	0.68 (0.64, 0.72)
Hosmer-Lemeshow test	p=0.455	p=0.753

* Number of participants with poor primary outcome *n*=768 (38%), ** Number of participants with poor secondary outcome *n*=698 (35,3%). ^1^Neck Disability Index, 0-100 (no-maximal disability).^2^A Numeric Rating Scale for arm pain (0-10). ^3^Pending medical claim/litigation against the Norwegian public welfare agency fund concerning disability pension or pending medical compensation claim/litigation against private insurance companies or the public Norwegian System of Compensation to Patients.^4^Area Under the operating Curve

Two risk matrices were developed for cases with low and high risk for non-success in neck disability, respectively. “Low-risk” was defined as having three out of the eleven risk factors in the prognostic model, while “high-risk” was defined as having six out of the eleven factors. The matrices are displayed in Table 4 and show that a low-risk individual the risk for non-success was 13%, whereas for a high-risk individual, the risk for non-success was 92%.

**Table 4:** Example of two cases with few or several positive predictors from the final prediction model for non-success in neck disability at 12 months. For each of the cases, the predicted probability has been calculated based upon the presence (yes) or absence (no) of each predictor

	Patient 1, few positive predictors	Patient 2, several positive predictors
Hard physical demands in work (vs computers/sitting still or light physical work)	No	Yes
High educational level (4 or more years of university) (vs high school or less than 4 years of university)	Yes	No
Pending litigation^1^ (vs none)	No	Yes
Previous neck surgery (vs not)	No	Yes
Duration of arm pain		
< 3 months	Yes	No
3–12 months	No	No
> 1 year	No	Yes
Baseline NDI^2^ score		
Low (0–40)	No	No
Medium (41–60)	Yes	No
High (> 60)	No	Yes
Anxiety or depression^3^	No	Yes
Probability	0.13 [0.12 to 0.15]	0.92 [0.91 to 0.94]

^1^Pending medical claim/litigation against the Norwegian public welfare agency fund concerning disability pension or pending medical compensation claim/litigation against private insurance companies or the public Norwegian System of Compensation to Patients. ^2^Neck Disability Index, 0–100 (no-maximal disability). ^3^Based on scoring “moderate” to “extremely” anxious or depressed in the item in EQ-5D-3L questionnaire

## Discussion

In this study, we found that more than one third of the patients reported non-successful outcome in neck disability or arm pain at 12-month follow-up after surgery for cervical degenerative radiculopathy. Patients with high risk for non-success in neck disability were characterized by physical demanding work, low level of education, pending litigation, previous neck surgery, duration of arm pain > 3 months and medium-to-high levels of baseline disability as well as anxiety/depression. The predictors for non-success in arm pain were foreign mother tongue, smoking, medium-to-high levels of baseline arm pain and all neck disability model predictors except for anxiety/depression.

The discriminative performance of the neck disability model was found to be good with an AUC of 0.78, whereas the arm pain model was slightly less accurate but still acceptable (0.68). A recent study on patients undergoing elective cervical spine surgery by Archer et al. reports slightly lower AUCs for a predictive model of worse NDI scores (0.64–0.69) and of worse arm pain scores (0.63–0.65) 1 year after intervention [2]. There is a large overlap of significant predictors between our two studies. For example, Archer et al. found that worsening of NDI and arm pain scores were significantly associated with longer symptom duration, workers’ compensation claims and higher baseline NDI — all of which are included in our two present models. In accordance with our results, Archer et al. found depression only to be significantly associated with worse NDI scores. Several other studies have shown a negative impact of mental health on outcomes after surgery for CDR [1, 11, 19, 23]. Further, Archer et al. found no association between worsening of scores and smoking or pre-operative pain level. In the present study, both factors were significantly associated with non-success in arm pain.

There exists conflicting evidence regarding gender and its impact on PROMs and other outcomes, such as length of hospital stay and complication rates after degenerative neck surgery [4, 18, 34]. Archer et al. found that female sex was among the predictors for worse neck disability scores but not for worse arm pain scores. In another multivariate analysis, Scerrati et al. found that female sex and two-level surgery (vs. one-level surgery) correlated with worse outcomes in NDI, as well as the use of postoperative collars, while BMI only was shown to be significant in an univariate analysis [34]. In the present model, neither gender, number of surgical levels nor obesity did show significant association with non-success in neck disability or arm pain. There are also conflicting results in literature regarding the impact of obesity on neck disability. For example, similar to our results, Sielatycki et al. found no correlation between a high BMI and cut-offs for several PROMs, including NDI [35], whereas Zhang et al. found that high BMI was associated with longer hospital stay, duration of surgery and higher postoperative complication rates [47].

The present study could not find that high age was a predictor of non-success in neither neck disability nor arm pain. This is supported by other multivariate studies [2, 29, 34]. Further, both comorbidity and ASA level only came out as significant predictors for non-success in the present univariate analyses but not in the final multivariate analysis. In a study of risk factors for failure to achieve a minimal clinically important difference (MCID) in NDI 12 months after surgery for cervical radiculopathy, a higher burden of comorbidity was found to be the most significant predictor [29]. Other studies have emphasized the significance of age and pre-operative functional status as a predictor of complications and mortality after cervical degenerative surgery [24, 28]. Since changing demographics are likely to significantly increase the age and frailty of those who seek operative care for cervical degenerative disease in the coming years, further research is warranted in relation to these aspects.

### Impact of findings

In the current healthcare environment, value-based thinking has brought more focus on quality and appropriateness of care. Also, as degenerative neck surgery is becoming increasingly safe and efficient, there is a need for more knowledge about which patients are not improving from surgery. The two present models can be used in a clinical setting to predict which patients will benefit from a surgical intervention and who will be better off being treated conservatively. To exemplify how these models can be used in a surgical practice, we produced a risk matrix constituted of two hypothetical patient scenarios for disability; one where the patient had several of the risk factors and another where the patient had only a few risk factors (Table 4). The patients with few predictors had low probability for non-success (0.13), while several predictors involved a high risk for non-success (0.92). According to our model, a patient with similar characteristics and symptomatology as described in the case study with few predictors should be reassured that surgery is a safe option in terms of improving from baseline arm pain and disability. Patients with a similar clinical picture as patient 2 with several positive predictors, on the other hand, should be counselled about alternative treatment strategies.

The present models can be further developed into a risk calculator to assess the probability of success or failure to achieve substantial change for every patient in a surgical practice. However, the model will first need to be further validated in other study populations. The feasibility of a risk calculator should also be evaluated.

### Strengths and limitations

An advantage of the present study is the large sample size of data captured in a national registry. NORspine was designed to prospectively capture important candidate predictors and PROMs prior to and during the year following surgery. The registry covers all the hospitals and private clinics conducting surgery on spinal disorders in Norway. A total of 78% of the operations are recorded in the registry [36]. Furthermore, our two models were well balanced with respect to the risk of overfitting, in particular the disability model which showed high accuracy with only seven included predictors.

In our study, we chose to include patients operated with both arthroplasty and fusion. The baseline characteristics and 12-month outcome data were similar between the groups, except for slightly higher NDI and NRS-NP scores for the arthroplasty patients at baseline, as well as a lower number of operated levels. There is no current consensus about the use of arthroplasty vs fusion in patients with degenerative cervical disease [8, 13, 16, 17, 42, 46]. One may question whether the results of the fusion group in our study can be generalized to the arthroplasty group since there are only 1% of arthroplasty patients in our cohort. Further studies are warranted to elucidate this issue.

Loss to follow-up was 35.6% at 12-month follow-up and could represent a selection bias. However, two recent Scandinavian spine registry studies based on similar cohorts have found that a loss to follow-up did not bias conclusions about treatment effects [22, 37].

Another potential limitation is related to the cut-off estimates of the applied PROMs. In the present study, we decided to use estimates of non-success instead of the concept of MCID. The main reason is that MCID often show to be less than measurement errors or estimates for smallest detectable change [43], making it difficult for a patient and/or a clinician to judge the clinical meaningfulness of these estimates. By using stricter estimates reflecting a substantial rather than minimal change, we argue that these cut-offs are better suited for use in the development of prediction models for non-success (or success).

The major limitation of the present study is that we did not externally validate the final models. External validation is necessary before these models can be further developed into a risk calculator used in clinical settings. Risk calculators may help inform discussions of surgical treatment options between surgeons and patients and lead to more accurate judgement of operative risk. In a clinical decision-making process, the probability of successful or non-successful outcomes of conservative treatment strategies also needs to be taken into consideration. The present study only investigated outcomes after surgical treatment and cannot be generalized to outcomes after non-surgical treatment options. Thus, there is a large need for exploring prediction models for both surgical and non-surgical treatment trajectories and outcomes.

## Conclusions

The final prediction model for non-successful outcome in neck disability 12 months after CDR surgery showed high discriminative performance, whereas the prediction model for arm pain was slightly less predictive. Based upon the two prediction models, individualized risk estimates can be made and used in shared decision-making with patients referred for surgical assessment. The models need to be externally validated and further tested in a clinical setting.
