# Correction to: Anterior surgical treatment for cervical degenerative radiculopathy: a prediction model for non‑success

**DOI:** 10.1007/s00701-024-06045-7

**Published:** 2024-03-25

**Authors:** Christer Mjåset, Tore K. Solberg, John‑Anker Zwart, Milada C. Småstuen, Frode Kolstad, Margreth Grotle

**Affiliations:** 1 Faculty of Medicine, University of Oslo, Oslo, Norway; 2 Department of Neurosurgery, Oslo University Hospital, Oslo, Norway; 3 Division of Clinical Neuroscience, Department of Research and Innovation, Oslo University Hospital, P.O. Box 4956, 0424 Oslo, Nydalen, Norway; 4 Institute of Clinical Medicine, The Arctic University of Norway, Tromsø, Norway; 5 Department of Neurosurgery and The Norwegian Registry for Spine Surgery (NORspine), The University Hospital of North Norway, Tromsø, Norway; 6 Department of Rehabilitation and Technology, Faculty of Health Science, Oslo Metropolitan University, St. Olavs Plass, P.O. Box 4, 0130 Oslo, Norway

**Correction to: Acta Neurochirurgica (2023) 165:145–157**

10.1007/s00701-022-05440-2

This study’s research protocol (#2014/1477) was assessed by the Regional Committees for Medical and Health Research Ethics (REK) to fall outside the scope of the Health Research Act and, accordingly, did not require approval from the ethical committees. The study was approved by the Data protection officer approval Oslo University Hospital (#18/10820).

